# Exploratory Analysis of Factors Associated With Pressure Injuries in Surgical Patients Assessed With the ELPO Scale

**DOI:** 10.1111/ijn.70170

**Published:** 2026-07-30

**Authors:** Manuela de Mendonça Figueirêdo Coelho, Beatriz Alves de Olivera, Thalia Alves Chagas Menezes, Viviane Mamede Vasconcelos Cavalcante, Ivina Maria Angelo Araújo, Manuela do Santos Gomes, Pedro Miguel Alves Rocha, Eliane Maria da Silva de Paula, Idevânia Geraldina Costa

**Affiliations:** ^1^ Federal University of Ceará Fortaleza Brazil; ^2^ Walter Cantídeo University Hospital Fortaleza Brazil; ^3^ Lakehead University Orillia Ontario Canada

**Keywords:** machine learning, patient safety, perioperative nursing, pressure injury, risk assessment

## Abstract

**Objective:**

The objective of this study is to analyse predictors associated with pressure injury occurrence and to explore the relationship between preventive resources and pressure injury outcomes in surgical patients assessed with the ELPO Scale.

**Methods:**

A retrospective cross‐sectional study was conducted in a university hospital surgical centre in Brazil. Data from 2698 adult surgical patients (2020–2022) were analysed. Variables included the ELPO score, surgical characteristics, preventive resources and clinical factors. Logistic regression, Random Forest and decision tree (CART) models were applied. Performance was assessed using AUC, sensitivity, specificity, positive predictive value, negative predictive value and F1 score.

**Results:**

The incidence of PI was 2.9%. Male sex (OR = 1.74), lateral surgical position (OR = 4.75) and use of a thermal mattress (OR = 2.11) were associated with increased risk. Surgical time ≥ 2 h was associated with lower odds of pressure injury occurrence in the adjusted model. A greater number of cushions was associated with reduced pressure injury occurrence in unadjusted analyses; however, this association was not consistently maintained after multivariable adjustment. Predictive models demonstrated limited discriminative performance (AUC≈0.60). The ELPO total score was the most influential predictor.

**Conclusions:**

ELPO‐based assessment and structured perioperative risk evaluation may support perioperative risk assessment and identification of patients at higher risk of pressure injury; however, the predictive models should be considered exploratory and require external validation.

## Summary

**What Is Already Known About This Topic?:**

- Perioperative pressure injuries remain a significant patient safety concern.
- Risk assessment tools such as the ELPO Scale are widely used in surgical settings.
- Predictive modelling in nursing remains underexplored in perioperative contexts.

**What This Paper Adds?:**

- This study develops logistic regression and Random Forest models to predict perioperative pressure injuries.
- The findings demonstrate limited discriminative performance in a large retrospective sample.
- The analysis identifies interacting clinical risk factors that are relevant to surgical positioning.

**Implications for Practice:**

- Exploratory predictive modelling may complement ELPO‐based risk assessment in future studies.
- Models should be interpreted as exploratory screening support tools.
- The findings reinforce targeted preventive positioning strategies.

## 1 Introduction

Pressure injuries (PIs) are frequent adverse events during the intraoperative period, with negative consequences for the patients and healthcare system. Recent studies indicate that the incidence of PI related to surgical positioning varies between 10% and 13%, especially in cardiac and orthopaedic surgeries, with risk factors associated with prolonged operative duration, medical devices and inadequate positioning (Altamimi et al. 2024).

In this context, the ELPO Scale—Risk Assessment Scale for Perioperative Pressure Injuries—was developed and validated in Brazil for risk assessment in the perioperative setting. With scores ranging from 7 to 35 points and good internal consistency and accuracy (sensitivity approximately 85% and specificity approximately 90%) (Salvini et al. 2024), it may support individualized perioperative risk assessment and hypothesis generation for future preventive strategies.

Despite reports on the prevalence of PI and associated clinical factors, there are gaps in the literature regarding the associations involving specific preventive interventions in patients screened using ELPO and the predictive performance of multiple combined variables (Souza et al. 2024).

The advancement of statistical models and machine learning has allowed the development of predictive approaches that integrate multiple clinical and administrative factors, offering additional support for clinical decision‐making for clinical decision‐making. An interpretable model with XGBoost (eXtreme Gradient Boosting), the most widely used machine learning algorithms, was used to predict PI caused by medical devices in the hospital setting, achieving Area Under the Curve (AUC) ≥ 0.90 and greater predictive power compared to traditional logistic regression (Qian et al. 2025).

Similarly, a nomogram was validated in neurosurgical patients, resulting in 77% sensitivity and 92% specificity with AUC‐ROC of 0.83–0.86 (Wang et al. 2025). In the ICU, XGBoost was applied with SHAP, a method used to explain the predictions of machine learning models. The SHAP analysis used data from the MIMIC IV (Medical Information Mart for Intensive Care, Version 4) database to predict PI in ventilated patients, with AUC of 0.739–0.797, highlighting variables such as sepsis, albumin, age and length of stay (Zheng et al. 2025).

Despite advances in perioperative PI prevention, important methodological and clinical gaps remain. Most studies have evaluated isolated clinical risk factors or validated traditional risk scales independently, without integrating structured perioperative assessment tools with predictive modelling approaches. In addition, evidence regarding the association between preventive positioning resources and PI outcomes within ELPO‐assessed populations remains limited and inconsistent.

Recent machine learning studies in hospital settings have demonstrated promising predictive performance for PI outcomes using complex datasets and high‐dimensional variables (Qian et al. 2025; Zheng et al. 2025). However, these studies were conducted primarily in intensive care or device‐related injury contexts and did not evaluate perioperative positioning injuries using a validated surgical risk assessment instrument such as the ELPO Scale.

Therefore, this study explores the integration of a validated perioperative risk assessment scale (ELPO) with interpretable predictive modelling techniques in order to identify potentially relevant predictors and generate hypotheses for future investigations.

## 2 Objective

The objective of this study is to analyse predictors associated with PI occurrence and to explore the relationship between preventive resources and PI outcomes in surgical patients assessed with the ELPO Scale.

## 3 Method

This retrospective cross‐sectional study was conducted using secondary data extracted from electronic medical records of a university hospital located in the capital of the state of Ceará, in the Northeast region of Brazil. The research was carried out by undergraduate students of the nursing programme, under the guidance and supervision of department faculty members, with the collaboration of service professionals from the hospital institution. This study was conducted and reported in accordance with the TRIPOD statement for prediction model studies (Collins et al. 2015).

The sample consisted of 3000 medical records of patients who underwent surgical procedures between July 2020 and November 2022. Data collection took place from January to October 2023. The study included the medical records of patients who underwent surgery during the defined period. Medical records of patients under 18 years of age were excluded. The final sample comprised 2698 medical records.

Data collection was carried out using a semistructured instrument, which was developed based on the variables present in safe surgery assessment tools. The investigated variables included the following predictors: surgery time, type of anaesthesia, type of surgical position, support surface, limb positioning, use of a thermal mattress, use of cushions, use of protective covers, presence of comorbidities, age, sex and total ELPO Scale score. The outcome included either presence or absence of PI. PI occurrence was defined according to institutional nursing documentation recorded during the postoperative hospitalization period. The outcome included the presence or absence of PI. PI occurrence was defined according to institutional nursing documentation recorded during the postoperative hospitalization period. PIs were identified during routine skin assessments performed by the bedside clinical nurses responsible for patient care during each shift. PI diagnosis and staging followed the National Pressure Injury Advisory Panel (NPIAP) classification system. For the purposes of this retrospective analysis, PI occurrence was considered when a PI was documented in the medical record after the surgical procedure, regardless of stage. The outcome was analysed as a binary variable (presence vs. absence of PI). Data completeness exceeded 95% for all variables, and no records required exclusion due to missing data. Therefore, no imputation procedures were applied. Data completeness exceeded 95% for all variables, and no records required exclusion due to missing data. Therefore, no imputation procedures were applied.

The data were organized in Microsoft Excel spreadsheets and later exported to the Statistical Package for the Social Sciences (SPSS) software, Version 23.0, and to Python (scikit‐learn), where the statistical analyses were performed. Initially, descriptive analyses (frequencies, means and standard deviations) and bivariate analyses (chi‐square tests, Fisher's exact test and *F* test) were applied to evaluate associations between the independent variables and the occurrence of PI. Then, multivariate logistic regression was performed to identify statistically significant predictive factors (*p* < 0.05). Variables considered clinically relevant based on prior literature and perioperative plausibility were evaluated in the multivariable model. Bivariate analyses were used as supportive exploratory procedures rather than as the sole criterion for predictor selection. Multicollinearity was assessed using variance inflation factors (VIFs), and no significant collinearity was detected among predictors. Model assumptions for logistic regression were verified prior to final model estimation. Given the retrospective design and low event rate, the modelling strategy was considered exploratory rather than confirmatory.

Additionally, a predictive model with the Random Forest algorithm was developed to evaluate the discriminative capacity of the variables in predicting PI. The Random Forest model was developed using 500 trees with default Gini impurity criteria for node splitting. Due to the low event rate, no oversampling techniques were applied, and class imbalance was handled through stratified sampling during data partitioning. Hyperparameter tuning was not performed because the analysis was exploratory and intended primarily for comparative interpretation rather than optimization.

Data were randomly split into training (70%) and testing (30%) sets using stratified sampling to preserve outcome distribution. Internal validation was performed using cross‐validation procedures. Model performance was primarily assessed using the area under the ROC curve (AUC), given the low event rate and imbalance in outcome distribution. To provide a more comprehensive assessment of predictive performance under class imbalance conditions, sensitivity, specificity, positive predictive value (PPV), negative predictive value (NPV), precision, recall and F1 score were also calculated. Calibration was explored descriptively by comparing predicted and observed probabilities across risk strata. Formal calibration statistics were not emphasized because the primary objective of the study was exploratory risk stratification rather than prediction model development for clinical implementation.

The relative importance of the variables was evaluated based on the internal criteria of the Random Forest model and presented graphically. In addition, a decision tree (CART) was constructed with the objective of structuring an interpretable clinical algorithm based on the most influential variables, using data processed in the R environment (Version 4.4.2).

Potential sources of bias include information bias inherent to retrospective medical record review and selection bias due to the single‐centre design.

### 3.1 Ethical Considerations

The study was approved by the Research Ethics Committee under Opinion No. 5.325.736 and Certificate of Presentation for Ethical Consideration (CAAE) No. 56064322.7.0000.5054. As this was documentary research with secondary data, the requirement for the Free and Informed Consent Form was waived, as provided for in CNS Resolution No. 466/2012.

## 4 Results

The sample consisted of 2698 patients who underwent surgical procedures. A predominance of females (52.9%) was observed, with a mean age of 47.8 years (SD = 20.3; range 18–96). Regarding surgical specialties, the most prevalent interventions were gynaecology (15.0%), general surgery (14.5%), mastology (12.4%), plastic surgery (7.3%) and vascular surgery (5.4%). Most procedures were performed in the supine position, with an average surgery time of 3 h (SD = 1.1).

All patients were assessed using the Risk Assessment Scale for Perioperative Pressure Injuries (ELPO), with a mean score of 16.5 points (SD = 2.9), indicating moderate to high risk of developing PI. The overall incidence of PI associated with surgical positioning was 2.9% (79/2698).

In the analysis of the association between the use of cushions and covers to prevent the occurrence of PI, no statistically significant associations were observed (Table 1). Although all patients who developed PI had used cushions, the use of this resource alone was not significantly associated with PI prevention (*p* = 0.086). Similarly, the use of dressings also did not show a significant association with the presence or absence of PI (*p* = 0.153), despite a slightly higher proportion of injuries among patients who did not use this type of material (5.1% vs. 2.8%).

**TABLE 1:** Data obtained through a safe surgery assessment instrument on the use of cushions and dressings. Fortaleza, Ceará, Brazil, 2023.

	Absence of PI		Presence of PI				
Variable	(*N*)	(%)	(*N*)	(%)	OR	95% CI	*p* ^a^
Use of cushions					1.031	0.990–1.073	0.086
No	94	100	0	0			
Yes	2296	97	72	3.0			
Use of dressings					0.541	0.230–1.274	0.153
No	112	94.9	06	5.1			
Yes	2278	97.2	66	2.8			

^a^ Chi‐square of independence.

In the comparison between the means of the continuous variables, it was observed that the mean ELPO Scale score was slightly higher among patients who developed PI (17.03 ± 3.13) compared to those who did not (16.45 ± 2.93), but without statistical significance (*p* = 0.095) (Table 2). Regarding the number of dressings used for prevention, there was also no statistically significant difference between the groups (*p* = 0.113), although the group with PI had a slightly higher mean (1.24 ± 0.77 vs. 1.13 ± 0.55).

**TABLE 2:** Analysis of data obtained through a safe surgery assessment instrument on ELPO scale score and number of dressings and cushions. Fortaleza, Ceará, Brazil, 2023.

	No PI mean	No PI SD	PI mean	PI SD	*F* test	*p* ^a^
ELPO score	16.45	2.93	17.03	3.13	0.002	0.095
Number of dressings	1.13	0.55	1.24	0.77	15.721	0.113
Number of cushions	5.72	2.24	5.07	1.83	5.582	0.014

Abbreviation: SD, Standard deviation.

^a^ *F* test.

The number of cushions used during surgery showed a significant association (*p* = 0.014), with a lower mean among patients who developed PI (5.07 ± 2.24) compared to those who did not (5.72 ± 1.83).

The number of cushions used showed a significant association in bivariate analysis (*p* = 0.014). However, after adjustment in the multivariate logistic regression model, this association did not remain statistically significant (*p* = 0.112).

A bivariate analysis was performed to identify the association between the studied variables and the occurrence of PI (Table 3).

**TABLE 3:** Analysis of data obtained through a safe surgery assessment instrument from patients who developed injuries after surgical procedure. Fortaleza, Ceará, Brazil, 2023.

	With PI					
Variables	(*N*)	(%)	OR	Min	Max	*p*
Sex			1.673	1.037	2.697	0.033^b^
Female	29	2.2				
Male	43	3.7				
			1.587	0.474	5.375	0.443^a^
Unconscious/intubated—no	38	4.1				
Unconscious/intubated—yes	03	6.4				
			1.043	0.515	2.113	0.908^b^
Anticoagulant use—no	10	3.2				
Anticoagulant use—yes	39	3.1				
			2.240	1.364	3.680	0.001^b^
Thermal mattress use—no	26	2.0				
Thermal mattress use—yes	42	4.3				
			0.541	0.230	1.274	0.153^b^
Dressings—no	06	5.1				
Dressings use—yes	66	2.8				
			1.031	0.990	1.073	0.112^a^
Cushions—no	00	0.0				
Cushions—yes	72	3.0				
			1.267	0.688	2.335	0.446^b^
ELPO score—low risk	59	2.8				
ELPO score—high risk	13	3.5				
			2.546	1.238	5.235	0.016^b^
Multilayer leg dressing—no	63	2.7				
Multilayer leg dressing—yes	09	6.6				
			1.831	0.895	3.744	0.105^b^
Transparent film dressing—no	63	2.8				
Transparent film dressing—yes	09	4.9				
			33.648	2.084	543.370	0.058^b^
Hydrocolloid dressing—no	71	2.9				
Hydrocolloid dressing—yes	01	50.0				

^a^ Fisher's exact test.

^b^ Chi‐square adherence.

The perioperative variables corresponding to ELPO domains were also explored in relation to PI occurrence. Significant associations were identified for surgical position (*p* = 0.007), surgical time (*p* = 0.003) and type of anaesthesia (*p* = 0.035). Patients positioned laterally presented the highest proportion of PIs (9.5%), and in the multivariable analysis, lateral positioning remained independently associated with increased odds of PI occurrence (OR = 4.75; 95% CI: 2.20–10.25; *p* < 0.001). Regarding surgical duration, procedures lasting ≥ 2 h showed lower odds of PI in the adjusted model (OR = 0.37; 95% CI: 0.15–0.87; *p* = 0.024), although this finding should be interpreted cautiously. General anaesthesia combined with regional anaesthesia demonstrated the lowest proportion of PIs (1.6%), whereas sedation and local anaesthesia presented higher proportions (5.4% and 5.1%, respectively). No statistically significant associations were observed for support surface, limb positioning, comorbidities or age categories in the exploratory analyses.

In the multivariate logistic regression analysis (Table 4), clinically relevant perioperative variables identified from prior literature and exploratory analyses were simultaneously evaluated to estimate independent associations with PI occurrence. The final model was statistically significant (*χ* ^2^[23] = 66.602; *p* < 0.001) and presented a Nagelkerke *R* ^2^ of 0.12, indicating that the set of variables accounted for a limited proportion of the variance in PI occurrence.

**TABLE 4:** Multivariate analysis by logistic regression of data obtained through a surgical safety assessment instrument for patients who presented PI after surgical procedures. Fortaleza, Ceará, Brazil, 2023.

Variable	Wald	df	Sig.	Exp(*B*)	Lower limit	Upper limit
Sex—female^a^	—					
Sex—male	4.557	1	0.033	1.744	1.047	2.908
Thermal mattress use—no^a^	—					
Thermal mattress use—yes	7.417	1	0.006	2.115	1.234	3.625
Cover use—no^a^	—					
Cover use—yes	3.299	1	0.046	0.400	0.163	0.984
Surgical position—supine^a^	—					
Surgical position—lateral	15.771	1	0.000	4.751	2.202	10.251
Surgical position—trendelenburg	0.000	1	0.999	0.000	0.000	
Surgical position—prone	0.655	1	0.418	1.241	0.506	3.045
Surgical position—lithotomy	0.223	1	0.637	1.241	0.506	3.045
Surgery time < 1 h^a^	—					
Surgery Time < 2 h	1.202	1	0.273	0.642	0.291	1.417
Surgery Time ≥ 2 h	5.107	1	0.024	0.370	0.156	0.876
Surgery Time > 4 h	0.020	1	0.889	0.937	0.377	2.327
Surgery Time > 6 h	1.517	1	0.218	0.419	0.105	1.674
Anaesthesia—local^a^	—					
Anaesthesia—sedation	1.134	1	0.287	3.294	0.367	29.546
Anaesthesia—regional	0.839	1	0.360	2.778	0.312	24.752
Anaesthesia—general	0.352	1	0.553	1.940	0.217	17.303
Anaesthesia—general + regional	0.034	1	0.854	1.244	0.121	12.737
Comorbidities—none^a^	—					
Comorbidities—vascular disease	0.120	1	0.729	0.894	0.475	1.683
Comorbidities—diabetes mellitus	2.132	1	0.144	0.567	0.265	1.214
Comorbidities—obesity or malnutrition	3.436	1	0.064	0.365	0.126	1.059
Comorbidities—pressure ulcer or neuropathy or DVT	1.050	1	0.306	2.208	0.485	10.047
Transparent film use—no^a^	—					
Transparent film use—yes	0.135	1	0.713	1.174	0.499	2.760
Multilayer cover use—no^a^	—					
Multilayer cover use—yes	3.232	1	0.072	2.102	0.935	4.723
Constant	53.883	1	0.000	0.028		

^a^ Reference category.

Hydrocolloid dressing was not retained in the final interpretative model due to sparse observations and unstable estimates. The variables that remained significantly associated with PI occurrence in the adjusted model were male sex (OR = 1.74; 95% CI: 1.047–2.908; *p* = 0.033), use of a thermal mattress (OR = 2.11; 95% CI: 1.234–3.625; *p* = 0.006), lateral surgical position (OR = 4.75; 95% CI: 2.202–10.251; *p* < 0.001) and cover use, which was associated with lower odds of PI occurrence (OR = 0.40; 95% CI: 0.163–0.984; *p* = 0.046). Surgical time ≥ 2 h also remained statistically significant (OR = 0.37; 95% CI: 0.156–0.876; *p* = 0.024).

To deepen the predictive capacity of the variables associated with the occurrence of PI related to surgical positioning, two models were adjusted: multivariate logistic regression and Random Forest.

Logistic regression achieved an AUC of 0.62, sensitivity of 0.75, specificity of 0.75, PPV of 0.10, NPV of 0.99 and an F1 score of 0.17. The Random Forest model achieved an AUC of 0.61. Overall, both models demonstrated limited discriminative performance, supporting exploratory risk stratification rather than clinical prediction. The high NPV and low PPV were consistent with the low incidence of PI in the study population. Descriptive calibration suggested reasonable agreement between predicted and observed probabilities across risk strata, although formal calibration statistics were not evaluated.

**FIGURE 1:**
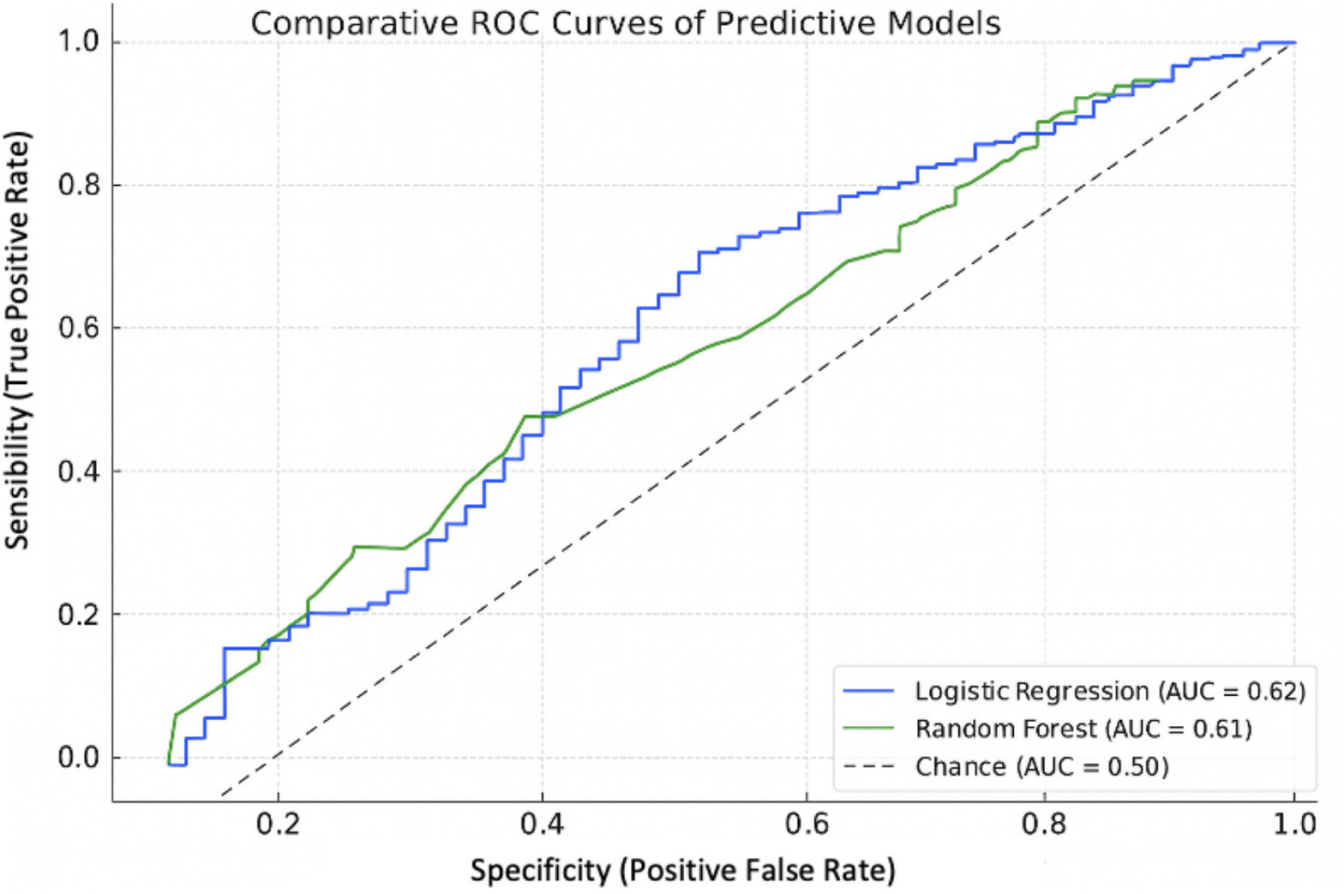
ROC curves of the predictive models tested for detecting PI related to surgical positioning.

Although the predictive models performed slightly above chance level, their discriminative capacity remains limited for individual clinical prediction. Therefore, these models should not be interpreted as standalone diagnostic tools but rather as exploratory analytical approaches capable of identifying clinically relevant patterns and supporting hypothesis generation for future prospective investigations (ROC curve; Figure 1).

**FIGURE 2:**
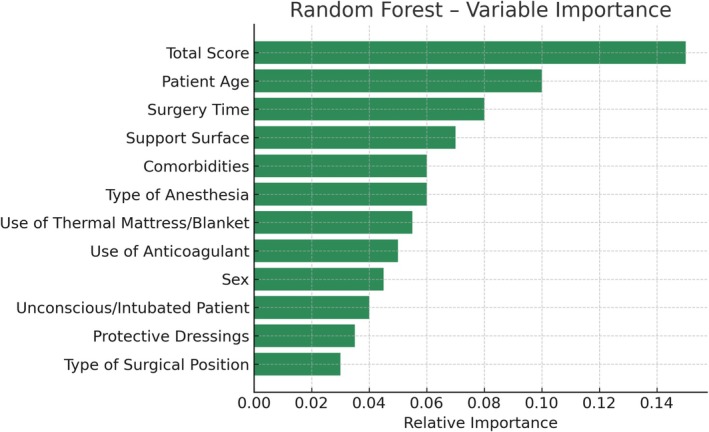
Relative importance of variables in the Random Forest model for predicting PI related to surgical positioning.

To complement the analysis of predictive performance, the relative contribution of each variable in the Random Forest model was examined. This approach enables the identification of the factors with the greatest influence on the classification of PI cases, as shown in Figure 2.

The variable importance analysis in the Random Forest model showed that the total ELPO scale score was the most relevant predictor of PI occurrence. Patient age emerged as the second most important variable, followed by surgery time and support surface. Comorbidities, type of anaesthesia, use of a thermal mattress or blanket, anticoagulant use and sex demonstrated intermediate contributions to model classification. Variables related to unconscious/intubated status, protective dressings and type of surgical position showed comparatively lower importance within the Random Forest model.

**FIGURE 3:**
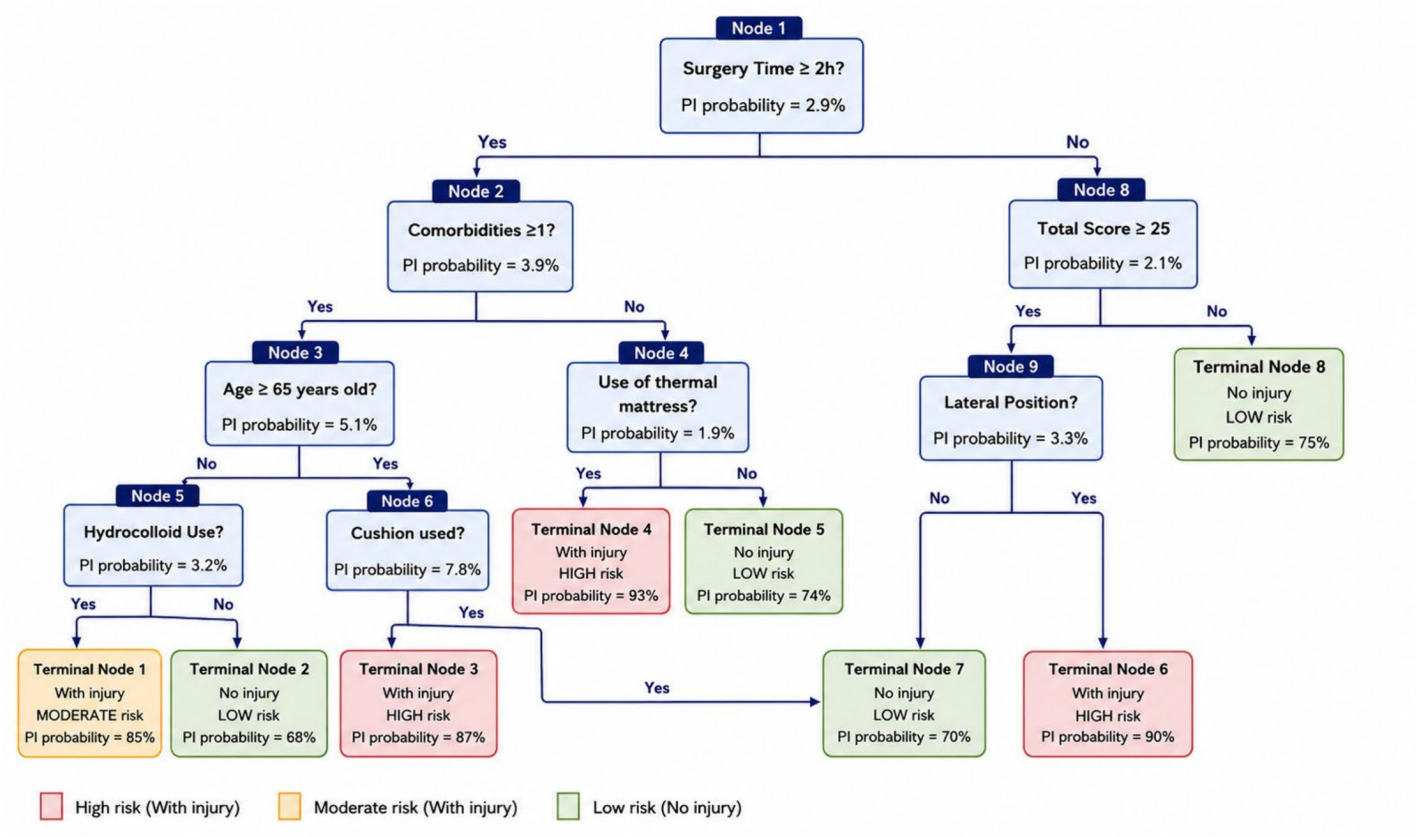
Classification and regression Tree (CART) for prediction of pressure injury in surgical patients. PI, pressure injury; PI probability, observed probability of pressure injury in the node.

Additionally, a decision tree (CART) was developed to provide an interpretable exploratory risk‐stratification framework based on the variables included in the predictive analysis (Figure 3).

The CART structure reflects recursive partitioning criteria and does not necessarily mirror the variable importance ranking obtained from the Random Forest model. The CART model supported clinical stratification; however, accuracy metrics should be interpreted cautiously due to the low event rate. Surgical time ≥ 2 h emerged within high‐risk interaction nodes, particularly when combined with other clinical variables. It is worth noting that the decision tree represents a predictive tool derived from observational data and should not be used as a definitive clinical guideline. Its use is recommended as a decision support tool, complementing individualized clinical assessment.

## 5 Discussion

The prevalence of PI observed in this study was 2.9%, a value lower than that reported in recent international investigations, especially in contexts of greater surgical complexity. For instance, in a study performed in a healthcare setting in Saudi Arabia, a prevalence of 13.6% of intraoperative PI related to medical devices was identified among patients undergoing cardiac and orthopaedic surgeries (Altamimi et al. 2024). Similarly, an incidence of 10.4% was observed in prolonged neurosurgical procedures, with a strong association with surgical time, age and lateral position (Wang et al. 2025).

In Brazil, studies conducted in public and teaching hospitals continue to report rates between 6% and 11% in the perioperative context (Peixoto et al. 2019; Salvini et al. 2024). The lower prevalence found in the sample of our study may be related to the systematic adoption of prevention protocols based on the ELPO scale, the predominance of short‐duration surgeries (average of 3 h) and the less invasive surgical profile, with lower frequency of long‐term procedures or high‐risk positions. These factors may have contributed to the lower occurrence of injuries, although underreporting and the use of secondary data should also be considered as possible limitations.

Among the statistically significant predictors for the occurrence of PI, male sex, lateral surgical position and use of a thermal mattress emerged as factors associated with increased risk, whereas surgical time ≥ 2 h was associated with lower odds of PI occurrence in the adjusted analysis. Male sex has been identified in previous studies as a potential risk factor, although underlying mechanisms remain uncertain (Wang et al. 2025).

The lateral position, in turn, is recognized as high risk for the development of PI, particularly in areas such as the trochanters and shoulders, due to the concentration of weight in specific points and the difficulty in redistributing pressure during the procedure (Altamimi et al. 2024). The use of a thermal mattress, although essential for the prevention of intraoperative hypothermia, has been related to a higher incidence of PI due to a potential increase in moisture and friction between the patient and the surgical table surface (Peixoto et al. 2019; Zheng et al. 2025).

The finding that surgical time ≥ 2 h was associated with lower odds of PI was unexpected and should be interpreted cautiously, as it contradicts established perioperative literature (Wang et al. 2025; Zheng et al. 2025). This result may reflect residual confounding, differences in surgical case mix, documentation practices or increased preventive vigilance during longer procedures. It is possible that patients undergoing prolonged surgeries received more comprehensive positioning protection and monitoring, whereas shorter procedures may have involved lower perceived risk and less rigorous preventive practices. Therefore, this association should not be interpreted as evidence that prolonged surgical time is inherently protective against PI.

Although surgical time ≥ 2 h was associated with lower odds of PI occurrence in the multivariate logistic regression model, the CART analysis identified prolonged surgical time as part of high‐risk profiles when combined with other variables, such as a lower number of cushions used and elevated ELPO scores. This difference likely reflects interaction effects captured by the decision tree model, highlighting the contextual and conditional nature of perioperative risk.

The performance of the ELPO scale in our study proved clinically relevant in predicting PI related to surgical positioning. The mean score was slightly higher among patients who developed PI (17.03) compared to those without any (16.45), although without statistical significance (*p* = 0.095).

However, in the Random Forest model, the total ELPO score stood out as the variable of greatest relative importance, reinforcing its role as a key component in risk classification. Such findings corroborate previous studies supporting ELPO as a useful tool for perioperative risk assessment (Lopes et al. 2016).

The identification of ELPO as the most influential variable does not demonstrate that the predictive models outperform the ELPO Scale when used alone. Comparative analyses between ELPO‐only performance and multivariable prediction models were beyond the scope of this exploratory study. Therefore, the findings should be interpreted as evidence of the relevance of ELPO within the predictive framework rather than proof of superior predictive performance. Future studies should directly compare the predictive performance of ELPO alone with multivariable and machine‐learning models in independent cohorts. At present, the findings support the relevance of ELPO as a key predictor within the modelling framework, but they do not establish whether multivariable models provide incremental predictive value beyond the ELPO Scale alone.

Nevertheless, it should be considered that the isolated use of risk scales may be insufficient, especially in high‐turnover environments or short‐duration procedures, in which factors such as team adherence and available time for intervention directly impact the associations involving preventive measures. Thus, although ELPO represents a valuable tool for initial screening, its application should be accompanied by complementary protocols and continuous clinical assessment, as part of a multifactorial prevention strategy (Prado et al. 2021).

The analysis of associations involving preventive measures revealed relevant findings regarding the use of cushions and protective dressings during surgical positioning. Although the isolated use of cushions did not show a statistically significant association with the occurrence of PI, it was observed that a greater number of cushions used was significantly associated with the absence of PI (*p* = 0.014). These findings suggest a possible association between greater cushion use and reduced PI occurrence in unadjusted analyses; however, this association did not remain statistically significant after multivariable adjustment. Therefore, the findings should be interpreted as exploratory and insufficient to establish effectiveness or causality (Prado et al. 2021).

On the other hand, findings regarding preventive dressings were mixed. Although the adjusted logistic regression model suggested an association between cover use and lower odds of PI occurrence (OR = 0.40; 95% CI: 0.16–0.98), individual dressing categories did not consistently demonstrate significant associations across analyses. Given the observational design, low event frequency and potential residual confounding, these findings should be interpreted cautiously and should not be considered evidence regarding the associations between preventive measures and PI outcomes.

The observed association between cover use and lower odds of PI occurrence may indicate that protective covers were preferentially applied in patients receiving more comprehensive preventive care. However, because preventive resources were not randomly allocated, this finding may also reflect residual confounding and differences in clinical decision‐making. Therefore, the association should not be interpreted as evidence of a causal association.

This finding is consistent with the literature that warns about the limitation of the isolated use of dressings as a preventive measure, recommending their application as a complement, and not a substitute, for pressure relief through positional strategies and support surfaces (Santamaria et al. 2019). In addition, nurses recognize the potential role of preventive dressings but emphasize that their use should be integrated with comprehensive prevention protocols, including repositioning for pressure relief, appropriate surfaces and skin integrity care, reinforcing the importance of a multifactorial approach based on clinical judgement (McMahon et al. 2024).

Similarly, another machine learning study for predictive models in hospital practice may prioritize sensitivity to reduce missed high‐risk cases (Padula et al. 2024). The evidence reinforces that, although logistic regression offers better interpretability, algorithms such as Random Forest have greater power to capture complex interactions and nonlinear variables, making them more suitable for clinical scenarios with large volumes and variability of data, such as the prediction of PI. The additional performance metrics provide further insight into model behaviour. Although discrimination was limited, the model achieved moderate sensitivity and specificity and a high negative predictive value. This finding suggests potential utility for identifying patients at lower risk of PI. Conversely, the low positive predictive value indicates that many patients classified as high risk would not develop PIs, a result largely influenced by the low incidence of the outcome. Consequently, the predictive models should be interpreted as exploratory tools for risk stratification rather than instruments for clinical decision‐making.

The construction of the decision tree (CART) in this study demonstrated its potential as a clinical tool for quick and visually intuitive screening of patients at high risk of developing PI. This practical applicability is supported by evidence indicating the satisfactory performance of the CART model in ICU patients, highlighting its usefulness in clinical practice (Xu et al. 2022).

A systematic review and meta‐analysis point out that models based on decision trees are promising in predicting PI, especially due to their interpretability, but require rigorous external validation before being widely used in hospital routines (Pei et al. 2023). This recommendation is consistent with the guidelines of the TRIPOD Statement, which emphasizes the need for external validation, calibration assessment and predictive performance evaluation in independent populations as essential steps before implementing models in diverse clinical contexts (Collins et al. 2015). Thus, although CART represents a promising approach, its application should be cautious and supported by additional multicentre studies in order to ensure its generalization and safety in care practice.

### 5.1 Limitations

Additional limitations should be acknowledged. The low incidence of PI resulted in outcome imbalance and limited statistical power for some subgroup analyses. Some preventive resource categories contained very small numbers of exposed patients, increasing the risk of unstable estimates and sparse data bias. Furthermore, the retrospective single‐centre design limits causal inference and external generalizability.

Future investigations should prioritize prospective studies, with a longitudinal design, that allow outcomes to be monitored over time and associations involving preventive interventions to be further investigated. Trials with control groups can strengthen the evidence regarding the association between protective measures and PI outcomes, especially those related to the use of dressings and support surfaces. In addition, it is essential to carry out external validation of the developed predictive models, in order to confirm their applicability in different clinical contexts. Finally, it is recommended to conduct cost‐effectiveness evaluation studies, aiming to identify the most viable and sustainable strategies for the prevention of PI in the surgical environment.

## 6 Conclusion

The findings show that male sex, lateral position and use of a thermal mattress were significant predictors for the occurrence of PI, whereas surgical time equal to or greater than 2 h was associated with lower odds of PI occurrence in the adjusted analysis. A greater number of cushions was associated with lower PI occurrence in unadjusted analyses; however, this association was not maintained after multivariable adjustment. The predictive models identified clinically relevant patterns; however, their discriminative performance was limited, and they should be interpreted as exploratory analytical tools rather than clinically applicable prediction models. These findings should be interpreted as exploratory and hypothesis generating. Although the predictive models identified clinically relevant patterns, their limited discriminative performance and lack of external validation preclude immediate clinical implementation. Further prospective studies and external validation are required.

The results of this study provide relevant evidence to inform nursing practices in the surgical context. The identification of significant predictors, such as surgical time, lateral position and perioperative positioning‐related factors, can guide more targeted interventions during patient positioning. In addition, the integration of predictive models and the decision tree into the care process represents an advancement in supporting clinical decision‐making for prevention of PI, allowing for faster and more well‐founded screening of patients at risk. These tools may contribute to future development of perioperative risk assessment strategies; however, external validation and prospective evaluation are necessary before any incorporation into clinical protocols can be recommended. Although predictive discrimination was limited, the findings reinforce the clinical relevance of structured perioperative risk assessment and positioning strategies in preventing PIs. External validation studies in independent surgical populations are necessary before these predictive approaches can be considered for broader clinical implementation.

## Author Contributions

*Conceptualization*: Manuela de Mendonça Figueirêdo Coelho, Viviane Mamede Vasconcelos Cavalcante and Idevânia Geraldina Costa. *Methodology*: Manuela de Mendonça Figueirêdo Coelho, Viviane Mamede Vasconcelos Cavalcante and Idevânia Geraldina Costa, Beatriz Alves de Olivera and Thalia Alves Chagas Menezes. *Formal analysis*: Manuela de Mendonça Figueirêdo Coelho. *Data curation*: Ivina Maria Angelo Araújo and Eliane Maria da Silva de Paula. *Investigation*: Beatriz Alves de Olivera, Thalia Alves Chagas Menezes, Ivina Maria Angelo Araújo, Eliane Maria da Silva de Paula and Pedro Miguel Alves Rocha. *Supervision*: Manuela de Mendonça Figueirêdo Coelho, Viviane Mamede Vasconcelos Cavalcante and Idevânia Geraldina Costa. *Writing – original draft*: Manuela de Mendonça Figueirêdo Coelho, Viviane Mamede Vasconcelos Cavalcante, Idevânia Geraldina Costa, Beatriz Alves de Olivera, Thalia Alves Chagas Menezes, Ivina Maria Angelo Araújo, Eliane Maria da Silva de Paula, Pedro Miguel Alves Rocha and Manuela do Santos Gomes. Writing – review and editing: Manuela de Mendonça Figueirêdo Coelho, Viviane Mamede Vasconcelos Cavalcante, Idevânia Geraldina Costa, Beatriz Alves de Olivera, Thalia Alves Chagas Menezes, Ivina Maria Angelo Araújo, Eliane Maria da Silva de Paula, Pedro Miguel Alves Rocha and Manuela do Santos Gomes.

## Funding

This research received a research grant from the Institutional Program for Scientific Initiation Scholarships of the Federal University of Ceará.

## Conflicts of Interest

The authors declare no conflicts of interest.

## Data Availability

The data that support the findings of this study are available from the corresponding author upon reasonable request. Data are not publicly available due to institutional and ethical restrictions.
